# Comparison of different microCT-based morphology assessment tools using human trabecular bone

**DOI:** 10.1016/j.bonr.2020.100261

**Published:** 2020-05-04

**Authors:** Lukas Steiner, Alexander Synek, Dieter H. Pahr

**Affiliations:** a Institute for Lightweight Design and Structural Biomechanics, TU-Wien, Vienna, Austria; b Department of Anatomy and Biomechanics, Karl Landsteiner University of Health Sciences, Krems, Austria

**Keywords:** Bone morphometry, Software comparison study, microCT, *μ*CT, Human trabecular bone, Trabecular thickness, Bone volume to total volume, Bone surface, Degree of anisotropy

## Abstract

MicroCT-based morphological parameters are often used to quantify the structural properties of trabecular bone. Various software tools are available for calculating these parameters. Studies that examine the comparability of their results are rare. Four different software tools were used to analyse a set of 701 microCT images from human trabecular bone samples. Bone volume to total volume (*BV*/*TV*), bone surface (*BS*), trabecular thickness (*Tb*. *Th*.) and degree of anisotropy (*DA*) were evaluated. *BV*/*TV* shows very low difference (−0.18 ± 0.15%). The difference in *BS* could be reduced below 5% if artificial cut surfaces are not included. *Tb*. *Th*. and *Tb*. *Sp*. show differences of maximal −12% although the same theoretical background is used. *DA* is most critical with differences from 4.75 ± 3.70% (medtool vs. Scanco), over −38.61 ± 13.15% (BoneJ vs. Scanco), up to 80.52 ± 50.04% (medtool vs. BoneJ). Quantitative results should be considered with caution, especially when comparing different studies. Introducing standardization procedures and the disclosure of underlying algorithms and their respective implementations could improve this issue.

## 1 Introduction

Quantitative bone morphology based on micro-computer tomography (microCT) allows the assessment of structural and mechanical properties from 3D trabecular bone structures. For example, bone volume to total volume (*BV*/*TV*) and degree of anisotropy (*DA*) have showed the ability to predict trabecular bone stiffness and yield strength Maquer et al. (2015); Musy et al. (2017). Variables such as trabecular thickness (*Tb*. *Th*.), trabecular number (*Tb*. *N*.) and bone surface (*BS*) are frequently used to quantify changes in bone remodelling simulation Schulte et al. (2013); Adachi et al. (2001).

Several software tools are available for the evaluation of morphological parameters that have been used in a number of studies Nazarian et al. (2007); Hosseini et al. (2017); Grassi (2016); Fatchiyah et al. (2015). However, there are no standardized methods for calculating these parameters. Information on the theory of the algorithms used in the tools can be found, but details of their implementations are unknown. Such software packages are treated more like black boxes.

So far, only a few studies tried to evaluate differences between certain software tools. A study by Salmon et al. (2015) compared results for the structure model index parameter (*SMI*) from two software tools (Bruker CTAn, BoneJ). They found a high correlation, but noted that the values were consistently different. Another study by Verdelis et al. (2011) compared the results from three *μ*CT systems (Scanco *μ*CT 35, Bruker Skyscan 1172 and GE Healthcare eXplore Locus SP). They found differences of up to 80% for *Tb*. *Th*., 150% for *Tb*. *N*. and 33% for bone surface to bone volume (*BS*/*BV*). The study was based on only nine samples of mouse bones and different CT systems were used for both scanning and data processing. It was unclear whether the differences were because of the different scanning devices or their respective software. Doube et al. (2010) compared results from CTan, BoneJ and Scanco's software. Only results for a single trabecular bone cube were reported and the study lacked statistics on the respective relations.

Given the lack of implementation details and the small sample sizes used in previous studies, it remains unclear whether *μ*CT-based morphometric parameters can be compared across studies if different software packages are used. The aim of this study is to fill these gaps by analysing a large set of 3D *μ*CT images from human trabecular bone samples with four different software tools and comparing the results.

## 2 Materials and methods

**Fig. 1:**
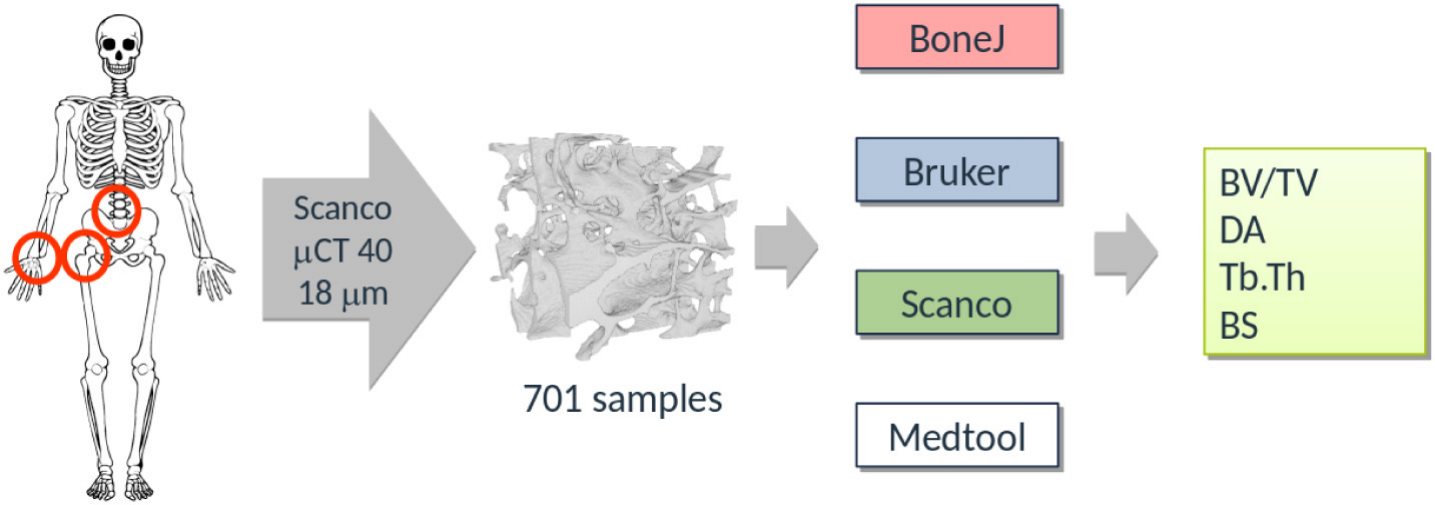
Graphical study overview: 701 segmented *μ*CT scans of trabecular bone samples were taken from an earlier study Gross et al. (2013). Four different software tools were used to calculate morphological parameters, which were finally compared.

**Fig. 2:**
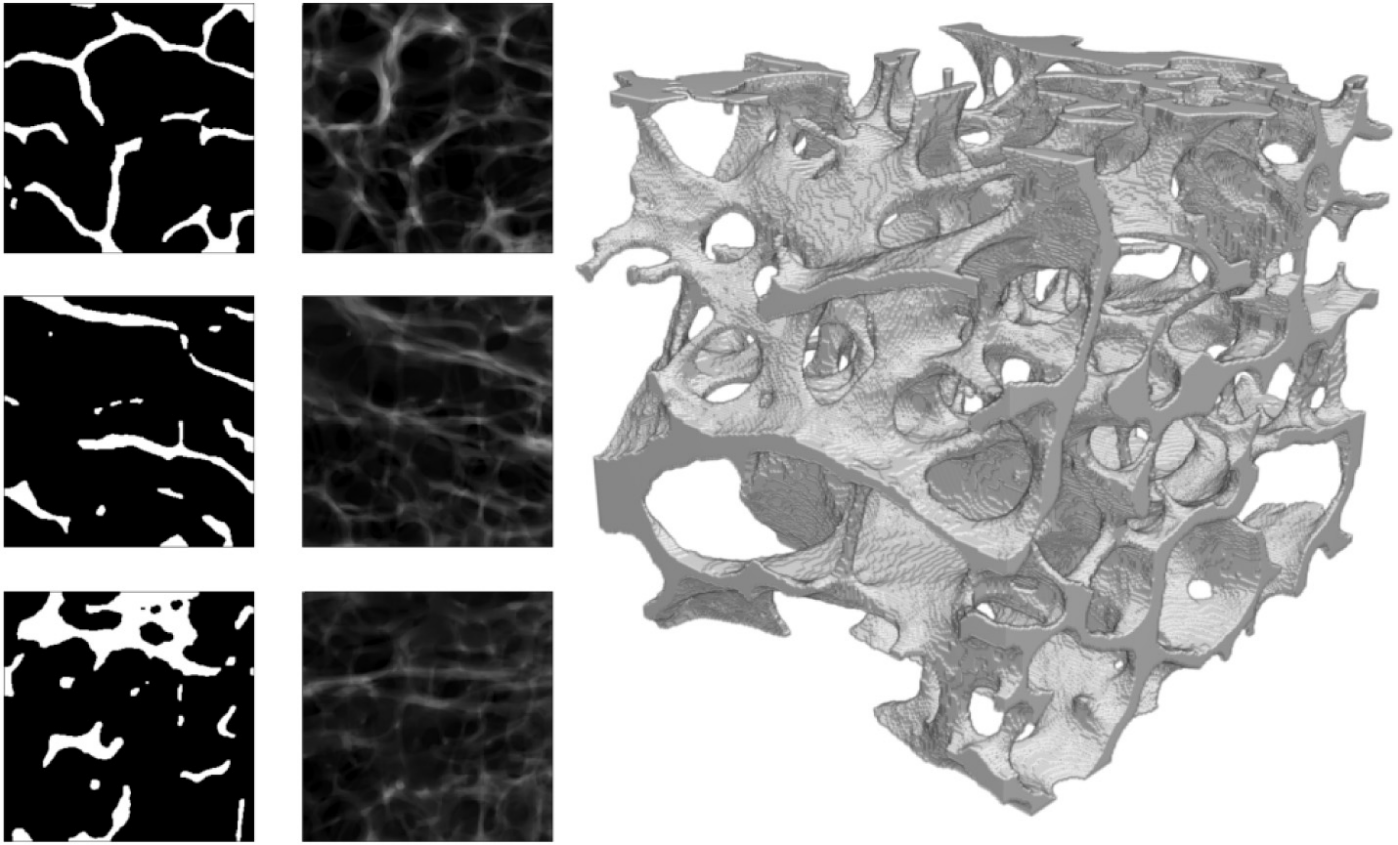
Midplanes (left), projections (center) and rendering (right) of one sample of the image set.

A graphical overview of the study can be found in Fig. 1. A set of 701 segmented *μ*CT images from a previous study was used. The samples were taken from several donors (male and female, age 44–82 years) at 3 different locations, namely radius, spine and femur. Scanning was done with a Scanco Medical *μ*CT 40. The original images were filtered with a Gaussian filter (*σ*=1.2,support=2), cropped and segmented with a single-level threshold. Unconnected sections were then removed Gross et al. (2013). A closing and an opening filter were applied (3 × 3 kernel). The final cubical regions of interest (ROI) consisted of 296×296×296 isotropic voxels with a side-length of 0.018 mm. Several views of one sample of the set are shown in Fig. 2.

One open source and three commercial software tools were used for evaluation: (1) The free, open-source ImageJ-plugin Schneider et al. (2012) BoneJ Doube et al. (2010) (v1.4.2; bonej.org), (2) the SKYSCAN CTan by Bruker (v1.17.7.2; Bruker Corporation, Billerica, USA), (3) the Scanco Image Processing Language IPL as part of the OpenVMS based evaluation platform by Scanco (v5.42; Scanco, Brüttisellen, Switzerland) and (4) the in-house script manager medtool (v4.3; Dr. Pahr Ingenieurs e.U., Pfaffstätten, Austria).

The following parameters were chosen for their frequent use in literature Chiba et al. (2012); Schwiedrzik et al. (2016); Hosseini et al. (2017): *BV*/*TV*, *BS*, *Tb*. *Th*. *Tb*. *Sp*. and *DA*. Original images are segmented prior to analysis to get the bone mask. Total volume (*TV*) is the volume of the cubical ROI. *BS* is determined by triangulating the bone mask and calculating the total area of the triangles (Lorensen and Cline (1987); Pahr (2018)). The bone volume (*BV*) is obtained by counting the bone mask voxels or computing the volume enclosed by *BS*. *Tb*. *Th*. is based on masked images and calculated by filling maximal spheres into the structure (Hildebrand and Rüegsegger (1997)). *Tb*. *Sp*. uses the same algorithm as *Tb*. *Th*. applied to the background of an image. *DA* is defined as the ratio between the maximum and the minimum eigenvalues of the mean intercept length (MIL) ellipsoid (Harrigan and Mann (1984)) and is determined by fitting an ellipsoid through a MIL distribution either from parallel test lines (Odgaard et al. (1997)) or from a projected triangulated *BS* (Hildebrand et al. (1999)). Table 1 summarizes the used methods. Default settings were used in almost all cases to minimize impact on results. An overview of these settings can be found in the Supplementary Table 1.

An analysis of the mean difference and the linear regression, including coefficient of determination *R*^2^, was performed. Statistics were calculated with python, using its scipy package (Jones et al. (2001-2019)). Linear regression and Bland-Altman plots Bland and Altman (1986) were used to display the results. Values obtained from medtool were used as reference for the plots.

**Table 1:** Evaluated parameters and their respective algorithms. Several methods and algorithms for the selected parameters are available. This table gives the cited methods and/or sources as given by the respective company. Sources: BoneJ - http://bonej.org, Bruker - CTAn manual, medtool - http://www.medtool.at, Scanco - *μ*CT Manual.

	BoneJ	Bruker	medtool	Scanco
*BV*/*TV*	Voxel counting	Lorensen and Cline (1987)	Voxel counting	Voxel counting
*BS*	Lorensen and Cline (1987)	Lorensen and Cline (1987)	Pahr (2018)	Hildebrand et al. (1999)
*Tb*. *Th*./*Tb*. *Sp*.	Hildebrand and Rüegsegger (1997)	Hildebrand and Rüegsegger (1997)	Hildebrand and Rüegsegger (1997)	Hildebrand and Rüegsegger (1997)
*DA*	Odgaard et al. (1997)	Odgaard et al. (1997)	Odgaard et al. (1997)	Hildebrand et al. (1999)

## 3 Results

**Fig. 3:**
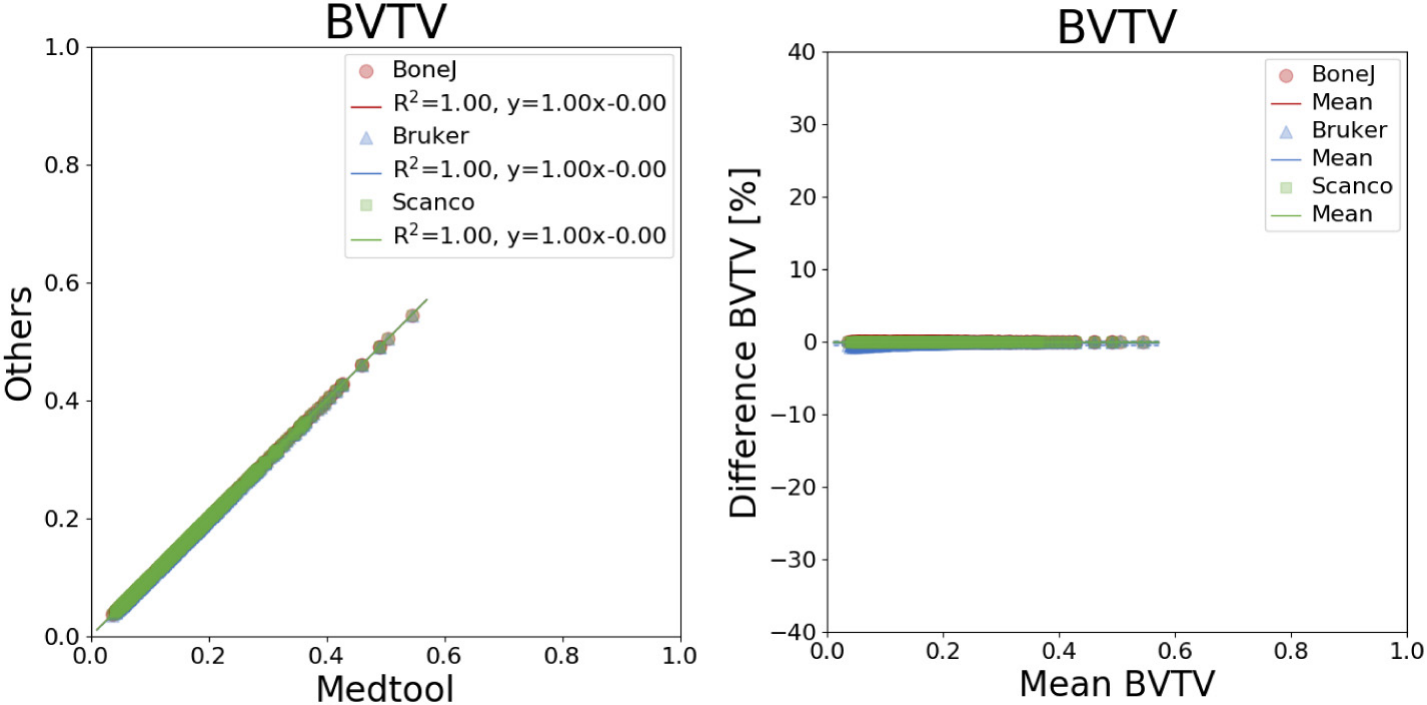
Linear regression (left) and Bland-Altman plot (right) for *BV/TV* with respect to medtool.

**Fig. 4:**
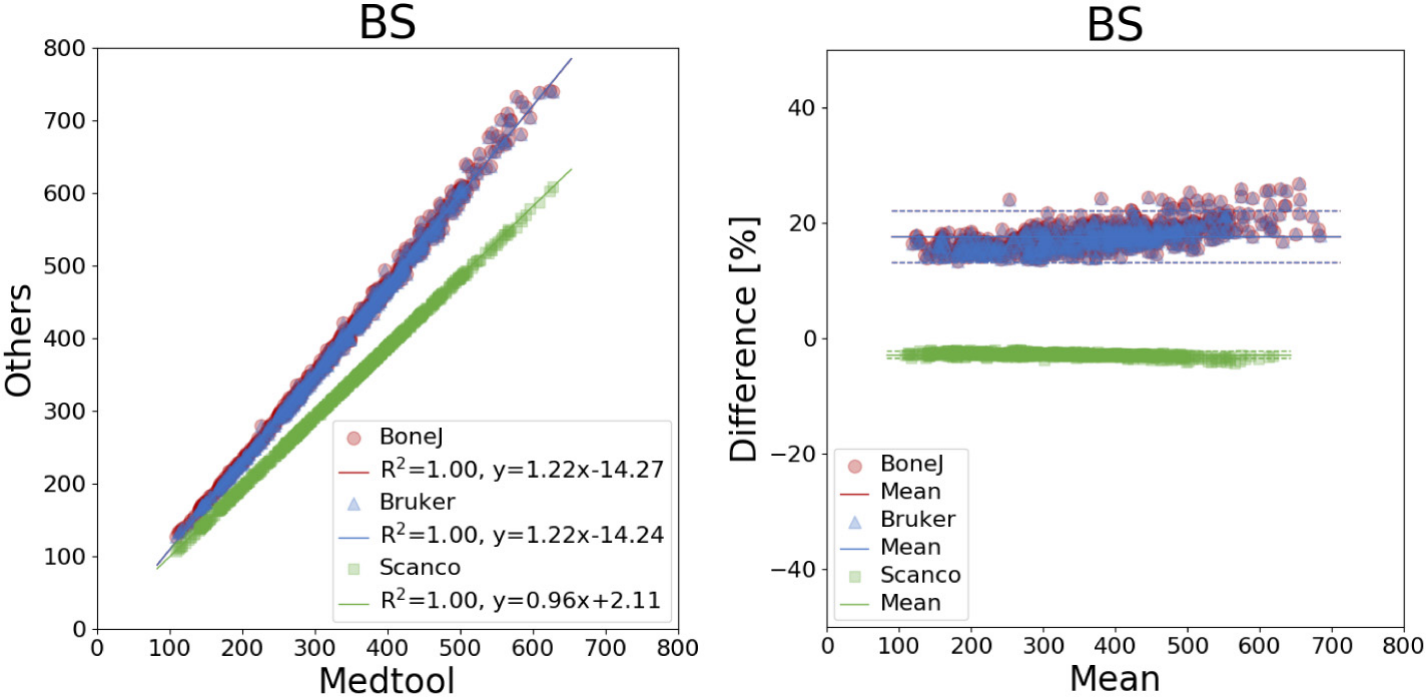
Linear regression (left) and Bland-Altman plot (right) for *BS* with respect to medtool.

**Fig. 5:**
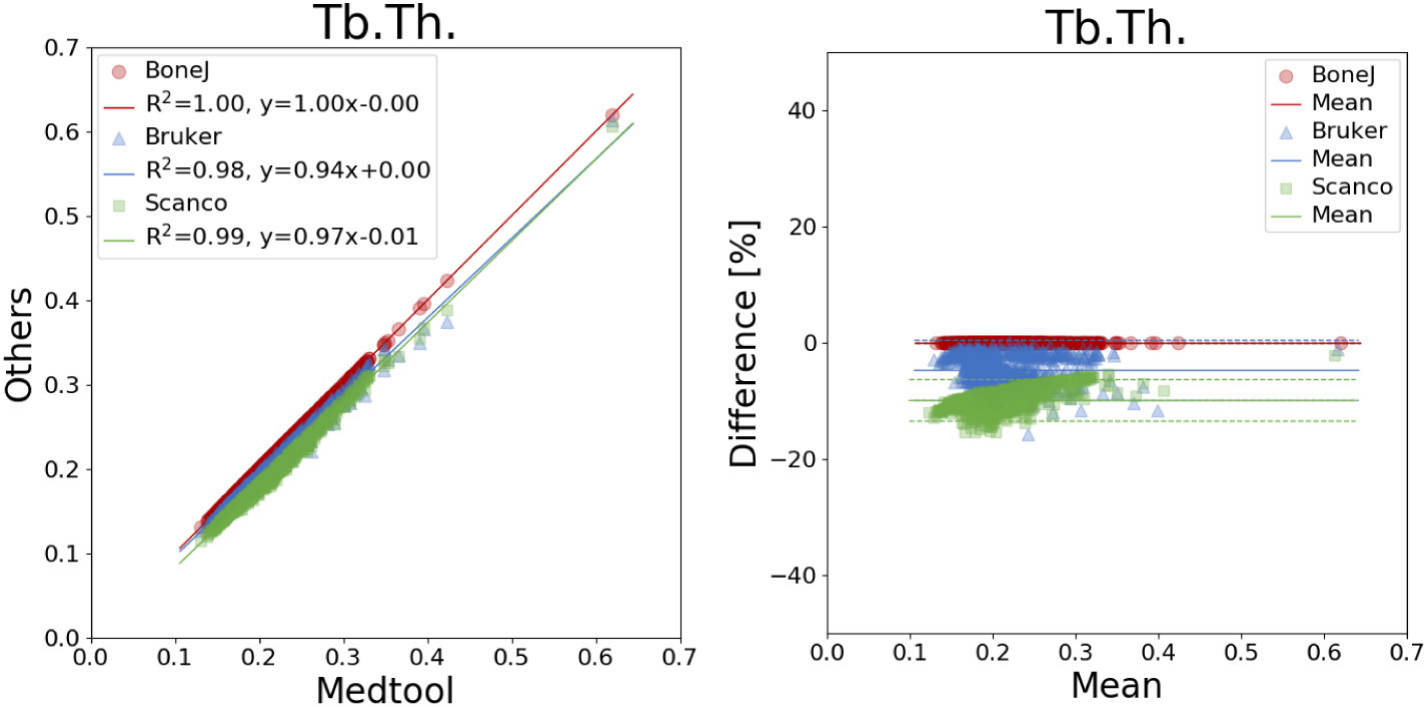
Linear regression (left) and Bland-Altman plot (right) for *Tb.Th.* with respect to medtool.

**Fig. 6:**
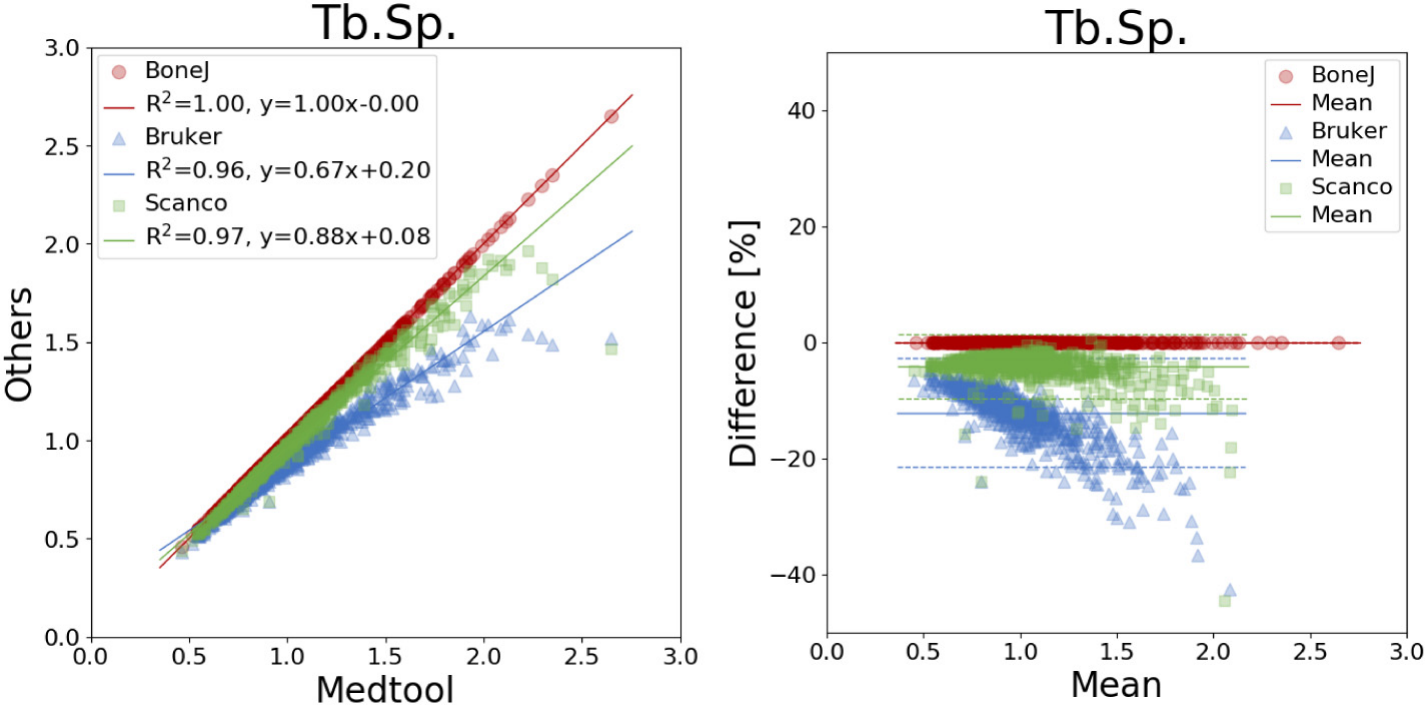
Linear regression (left) and Bland-Altman plot (right) for *Tb.Sp.* with respect to medtool.

**Fig. 7:**
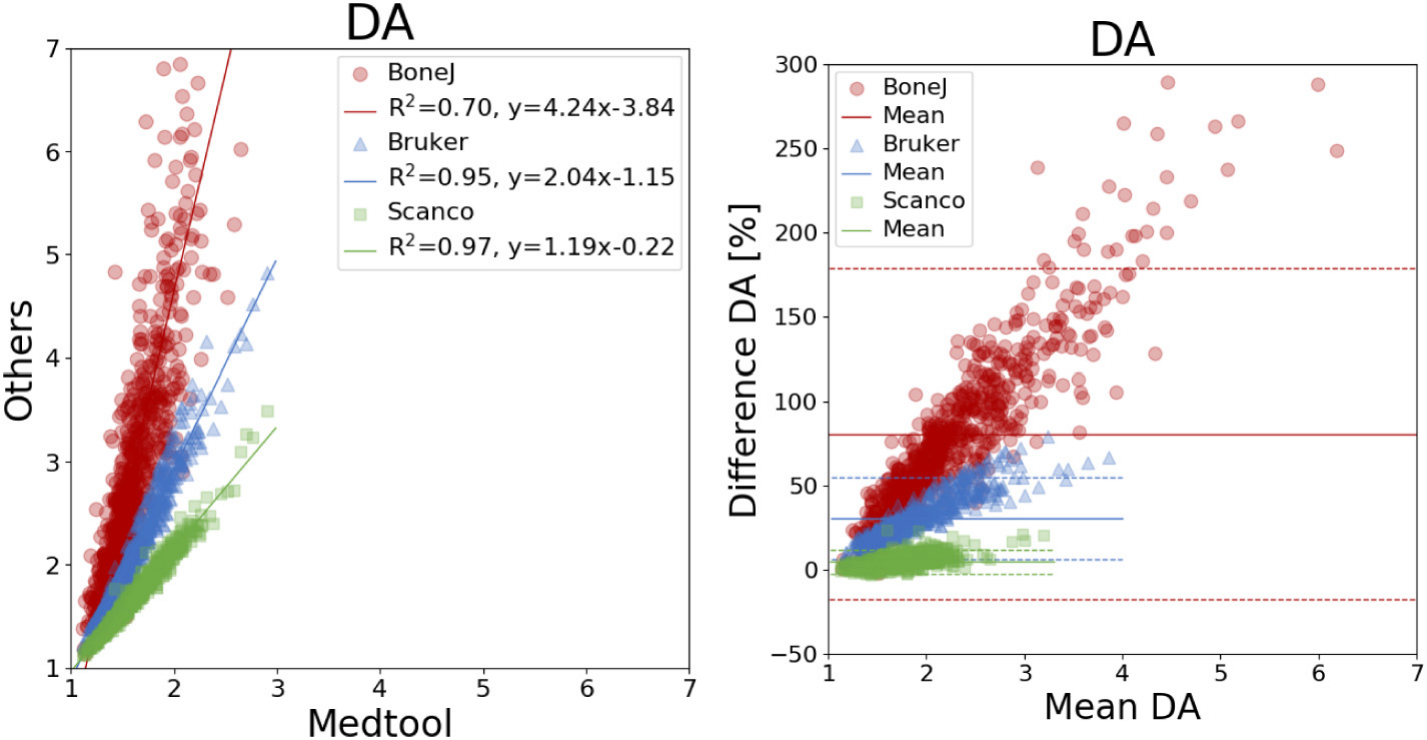
Linear regression (left) and Bland-Altman plot (right) for *DA* with respect to medtool.

An overview of all mean differences is given in Table 2 and coefficients of determination are listed in Table 3. The corresponding plots are shown in Fig. 3, Fig. 4, Fig. 5, Fig. 6, Fig. 7. The mean differences are shown as solid lines in the Bland-Altman plots, while dashed lines show a standard deviation of ± 1.96. High correlations were found for almost all parameters except the *DA* values of BoneJ. Mean differences, with the exception of *BV*/*TV*, are considerable.

**Table 2:** Overview of mean differences and standard deviations.

diff ± std. [%]	medtool - BoneJ	medtool - Bruker	medtool - Scanco	BoneJ - Bruker	BoneJ - Scanco	Bruker - Scanco
*BS*	17.60 ± 2.26	17.64 ± 2.26	−2.87 ± 0.33	0.03 ± 0.02	−17.37 ± 1.79	−17.40 ± 1.78
*BV*/*TV*	0.00 ± 0.00	−0.18 ± 0.15	0.00 ± 0.00	−0.18 ± 0.15	0.00 ± 0.00	0.18 ± 0.15
*Tb*. *Th*.	−0.03 ± 0.01	−4.71 ± 2.65	−9.95 ± 1.82	−4.67 ± 2.66	−9.92 ± 1.82	−5.46 ± 2.07
*Tb*. *Sp*.	−0.01 ± 0.01	−12.11 ± 4.78	−4.15 ± 2.81	−12.11 ± 4.79	−4.14 ± 2.81	9.28 ± 4.65
*DA*	80.52 ± 50.04	30.66 ± 12.46	4.75 ± 3.70	−24.19 ± 14.12	−38.61 ± 13.15	−19.30 ± 5.97

**Table 3:** Overview of coefficients of determination.

R^2^	medtool - BoneJ	medtool - Bruker	medtool - Scanco	BoneJ - Bruker	BoneJ - Scanco	Bruker - Scanco
BS	1.00	1.00	1.00	1.00	1.00	1.00
*BV*/*TV*	1.00	1.00	1.00	1.00	1.00	1.00
*Tb*. *Th*.	1.00	0.98	0.99	0.98	0.99	0.99
*Tb*. *Sp*.	1.00	0.96	0.97	0.96	0.97	0.99
*DA*	0.70	0.95	0.97	0.70	0.74	0.96

It was found that *BV*/*TV* is almost the same in all software tools, with differences ranging from −0.18 ± 0.15% (medtool vs. Bruker) to 0.18 ± 0.15% (Bruker vs. Scanco).

Mean differences of *BS* were up to 18% (Fig. 4). The largest deviations were found between the results of BoneJ and Bruker with respect to medtool: 17.60 ± 2.26% for BoneJ and 17.64 ± 2.26% for Bruker, respectively. BoneJ and Bruker showed differences of −17.37 ± 1.79% and −17.40 ± 1.78% to Scanco.

Mean differences of up to −10 ± 2% were found for *Tb*. *Th*. (Fig. 5), with all coefficients of determination larger than 0.98. The mean difference was smallest with −0.03 ± 0.01% between medtool and BoneJ, and largest between medtool and Scanco with −9.95 ± 1.82% as seen in Fig. 5.

Mean differences of up to −12 ± 5% were found for *Tb*. *Sp*. (Fig. 6), with all coefficients of determination larger than 0.96. The mean difference was smallest with −0.01 ± 0.01% between medtool and BoneJ, up to −12.11 ± 4.78% between medtool and Bruker as seen in Fig. 6.

*DA* showed differences up to 81% (Fig. 7), with a standard deviation of 50%. Here, BoneJ showed the highest difference with respect to medtool with 80.52 ± 50.04%, followed by BoneJ and Scanco with −38.61 ± 13.15%, down to BoneJ and Scanco with −4.14 ± 2.81%. Coefficients of determination ranged from 0.70 to 0.97 as seen in Table 2.

## 4 Discussion

In this study, four frequently used software tools for *μ*CT morphology assessment were used to analyse a large set of trabecular bone images. The calculated parameters were compared to determine the differences between the algorithms of the software tools. High correlations were found in almost all cases. However, mean differences ranged from 0% for *BV*/*TV* up to 81 ± 50% for *DA*.

The comparison of our results with Verdelis et al. (2011) shows that the variations reported there cannot be entirely attributed to scan- and segmentation-related issues. The values reported in Doube (2015) for a single trabecular bone cube, analysed with three software tools, showed similar tendencies to those found in this work. There, the differences for *BV*/*TV*, *Tb*. *Th*. and *DA* were up to 6%, 8%, and 30% respectively. The bone sample was scanned and analysed by Scanco and Bruker and the results are subject to the influence of the scanning and software evaluation. Our results show that a considerable amount of these differences comes from the used software tools only. They are based on an analysis of the same images with different software programs. The differences found are therefore not attributable to hardware, scanning or segmentation.

*BV*/*TV* was the only parameter with almost no mean differences between all software tools. This was expected because of the simple nature of its calculation. A small mean difference was found between medtool and Bruker, most likely because Bruker's software computes the *BV* from triangulated surfaces. The accuracy and precision of *BV*/*TV* makes it a very reliable parameter.

Large mean differences were found between all tools regarding *DA*. All methods are based on a fitted MIL ellipsoid. However, while Scanco uses a MIL distribution calculated from projected triangulated surfaces, all others are based on parallel test lines. Interestingly, medtool and Scanco only show a 5% difference of the mean, while Bruker and BoneJ show differences of 30% and 80% with respect to medtool, respectively. A possible explanation for this is that the calculation of the MIL distribution includes several parameters that are not known (test line distance, orientation, etc.), and the normalization of the eigenvalues can also be different. These results are especially critical due to the use of this parameter for the prediction of mechanical properties of trabecular bone Maquer et al. (2015); Musy et al. (2017) and in paleontological research Tsegai et al. (2013). These differences should be held in mind when comparing results from various publications using *DA*.

**Fig. 8:**
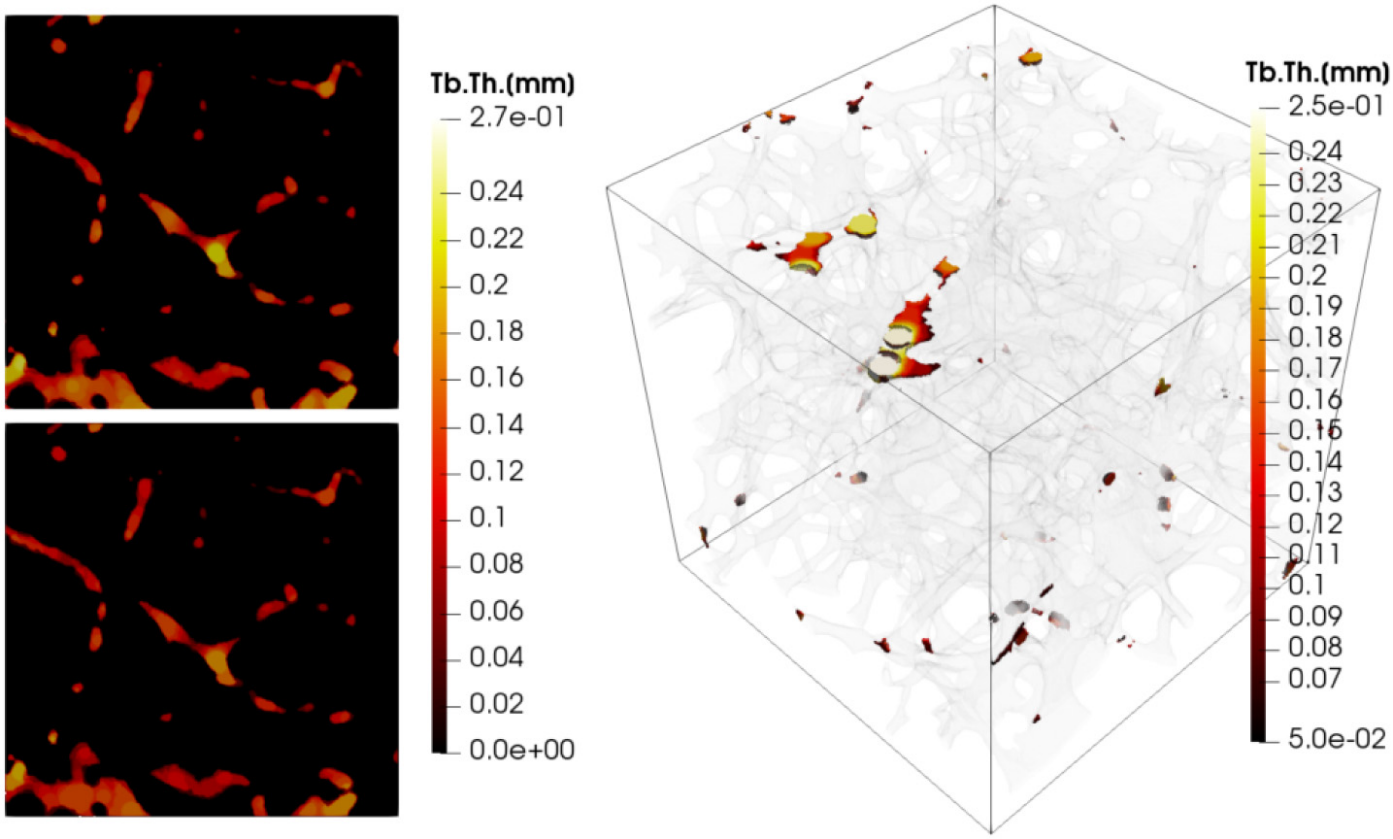
Thickness maps showing the Tb.Th. distribution for a selected example. medtool (top left) and Scanco (bottom left) sections are shown. Both are largely similar whereas Scanco values are slightly lower. Differences are particularly evident at the edges of the image. The right side shows Tb.Th. differences over 0.05 mm in color and the underlying geometry is shown transparently.

The differences in the results for *BS* were around 18% for Bruker and BoneJ, when compared to others, and − 3% for Scanco vs. medtool, while the coefficients of determination were 1.00. The large deviations of the Bruker and BoneJ results, with respect to medtool and Scanco, indicate a systematic deviation. Additional evaluations were carried out with medtool, where surface areas at the ROI boundaries were included in the calculation. The differences to BoneJ and Bruker dropped to about 5% while the difference to Scanco increased to −13%. Coefficients of determination all remained at 1.00. Scanco explicitly states in their *μ*CT manual that “artificial boundaries at the edge of cubic… volumes of interest are not counted in the calculation”. No such information could be found for the other two companies but it seems that these surfaces are included. Even methods such as marching cubes can lead to inconsistent results. Results for *Tb*. *Th*. and *Tb*. *Sp*. again showed high correlations between all implementations, with all *R*^2^*s* higher than 0.96. Although all algorithms are said to be based on Hildebrand and Rüegsegger (1997), only the results of medtool and BoneJ were found to be almost equal, as the source of BoneJ is open and has been reimplemented very similar in medtool. The mean differences of up to −12% between the other tools in turn indicate variations in their implementations. Thickness maps showing the assigned thickness of each voxel in an image were created with medtool and Scanco to get a better insight into these variations. The images are shown in Fig. 8. It can be seen that the results are largely similar, with slightly higher results from medtool. However, there are big differences especially at the edge of the model, because the algorithm of Scanco apparently does not include voxels in this region. The used version of Bruker's CTan did not provide an option to generate thickness maps. The results of BoneJ were very similar to those of medtool. In summary, *Tb*. *Th*. and *Tb*. *Sp*. should be used with caution.

The study is limited to the use of human trabecular bone samples, scanned at one *μ*CT system with a fixed resolution of 0.018 mm. Other micro-structures, e.g. of animals, and scans at different *μ*CT systems and/or resolutions, can lead to different outcomes.

Only real bone samples were analysed. The scope of the study is to highlight differences in the tools' results. Future studies could focus on the differences in the respective implementations and the definition of test structures as ground truth.

In summary, it could be shown - based on a huge set of human trabecular bone samples - that the quantitative CT morphology strongly depends on the investigated parameters and used software tools. In future, standardization will be needed if the results of different studies based on different assessment methods are to be compared.

The following is the supplementary data related to this article.

Supplementary Table 1 Parameter settings as used in the study. No data is given if no options were available. Supplementary Table 1

## CRediT authorship contribution statement

**Lukas Steiner:**Software, Investigation, Formal analysis, Writing - original draft, Visualization.**Alexander Synek:**Visualization, Writing - review & editing.**Dieter H. Pahr:**Software, Resources, Writing - review & editing, Visualization, Conceptualization, Supervision.

## Declaration of competing interest

Dieter H. Pahr is CEO of Dr. Pahr Ingenieurs e.U., which develops and distributes medtool.
