# Supplementary material for: Comparison of different microCT-based morphology assessment tools using human trabecular bone
Source: Bone Rep. 2020 May 4;12:100261. doi: 10.1016/j.bonr.2020.100261 (PMC7235944; doi:10.1016/j.bonr.2020.100261)
Supplement: Supplementary Table 1 — Parameter settings as used in the study. No data is given if no options were available. [file mmc1.pdf]

	**BoneJ**	**Bruker**	**medtool**	**Scanco**
BV/TV	Algorithm: Voxel	-	-	Voxel
BS	Resampling: 1	-	Surface: vxi_mi	ip_sigma: 0.0<br>ip_support: 0<br>ip_threshold: 50<br>nr_ave_iter: 2<br>t_dir_radius: 2<br>size_image: 512<br>scale_image: 0.7<br>edges: false
Tb.Th./<br>*Tb.Sp.*	-	-	-	peel_iter: -1<br>roi_radius_factor: 10000<br>ridge_epsilon: 0.9<br>assign_epsilon: 1.8
DA	Automode<br>Vectors: 50000<br>Min spheres: 100<br>Max spheres: 2000<br>Tolerance: 0.005	Adaptive spacing: 200<br>Number of<br>orientations: 128<br>restrict thickness calculation within VOI	Normalization: trace	ip_sigma: 0.0<br>ip_support: 0<br>ip_threshold: 50<br>nr_ave_iter: 2<br>t_dir_radius: 2<br>size_image: 512<br>scale_image: 0.7<br>edges: false

Supplementary Table 1: Parameter settings as used in the study. No data is given if no options were available.
